# Effects of Tip Sonication Parameters on Liquid Phase Exfoliation of Graphite into Graphene Nanoplatelets

**DOI:** 10.1186/s11671-018-2648-5

**Published:** 2018-08-17

**Authors:** Xinzhi Cai, Zeyi Jiang, Xinru Zhang, Xinxin Zhang

**Affiliations:** 1 School of Energy and Environmental Engineering, University of Science and Technology Beijing, Beijing, 100083 China; 2 Beijing Key Laboratory for Energy Saving and Emission Reduction of Metallurgical Industry, University of Science and Technology Beijing, Beijing, 100083 China; 3 Beijing Engineering Research Center of Energy Saving and Environmental Protection, University of Science and Technology Beijing, Beijing, 100083 China

**Keywords:** Graphene nanoplatelets (GNPs), Liquid phase exfoliation (LPE), Sonication parameters, Sonication energy input

## Abstract

Graphene nanoplatelets (GNPs) can be produced by exfoliating graphite in solvents via high-power tip sonication. In order to understand the influence of tip sonication parameters on graphite exfoliation to form GNPs, three typical flaked graphite samples were exfoliated into GNPs via tip sonication at power of 60, 100, 200, or 300 W for 10, 30, 60, 90, 120, or 180 min. The concentration of GNP dispersions, the size and defect density of the produced GNPs, and the sedimentation behavior of GNP dispersions produced under various tip sonication parameters were determined. The results indicated that the concentration of the GNP dispersions was proportional to the square root of sonication energy input (the product of sonication power and time). The size and *I*_D_/*I*_G_ values (determined by Raman spectrum) of GNPs produced under various tip sonication powers and times ranged from ~ 1 to ~ 3 μm and ~ 0.1 to ~ 0.3, respectively, which indicated that all the produced GNPs were of high quality. The sedimentation behavior of GNP dispersions showed that the dispersions were favorably stable, and the concentration of each GNP dispersion was ~ 70% of its initial concentration after sedimentation for 96 h. Moreover, the TEM images and electron diffraction patterns were used to confirm that the produced GNPs were few-layer. This study has important implications for selecting the suitable tip sonicating parameters in exfoliating graphite into GNPs.

**Electronic supplementary material:**

The online version of this article (10.1186/s11671-018-2648-5) contains supplementary material, which is available to authorized users.

## Background

Graphene is a kind of two-dimensional layered material with an exceptionally high Young’s modulus of ~ 1.0 Tpa, ultrahigh thermal conductivity of ~ 5000 W/(m · K), high transmittance of 97.7%, high intrinsic mobility of ~ 200,000 cm^2^/(V · s), and extremely high resistance to gas permeation [1–3]. Because of these outstanding properties, graphene has great potential for many applications including sensors, electronic devices, advanced polymer nanocomposites, energy storage, solar cells, smart coatings, ultrafast lasers, catalysis, and biological labeling [2, 4–6]. The unique properties and potential applications have led to researchers exploring promising methods to produce graphene in the past several years.

To date, a series of methods have been developed to produce graphene, such as micromechanical cleavage [7], reduction of graphene oxide [8, 9], chemical vapor deposition (CVD) [10], and liquid-phase exfoliation (LPE) [11–14]. Micromechanical cleavage can be used to prepare high-quality large-area GNPs but has the disadvantages of low production yield and poor throughput. Reduction of graphene oxide is widely used to produce GNPs; however, the reduction process does not remove all of the oxygen functional groups. Thus, the GNPs produced by reduction of graphene oxide still retain a high defect density, which degrades their properties. CVD is a promising method for large-scale production of monolayer or few-layer graphene with high quality; however, the method requires harsh chemical reaction conditions, such as high temperature and vacuum, which may increase the costs and cause safety problems. LPE was first carried out by Coleman et al. [11] by sonicating graphite in organic solvents using a bath sonicator. Because of its low cost, simplicity, and potential for large-scale production, LPE has attracted much attention from many researchers and become a promising method to produce GNPs.

Generally, the LPE process involves three steps [15], i.e., dispersing graphite in an appropriate solvent, exfoliating graphite into GNPs by different techniques, and then purifying the GNPs. Many researchers have made efforts to screen promising solvents and develop potential exfoliation techniques. Regarding solvent screening, more than 60 solvents have been used to exfoliate graphite to date, including various organic solvents [16], solvents with low boiling points [17, 18], surfactant solutions [12, 19], ionic liquids [20], polymer solutions [21], and amphiphilic biomolecule solutions [22]. In addition, to predict the good solvents, the surface tension theory [11] and Hansen solubility parameters [16] have been used to explore the mechanism of graphite exfoliation.

In terms of exfoliation techniques, sonication [23–26], high-shear mixing [27, 28], ball milling [29], and high-pressure homogenization [30] have been employed in LPE. Among these methods, sonication is widely used in LPE, which include two categories, i.e., bath sonication and tip sonication. Bath sonication is a convenience and low-cost method to exfoliate graphite [31]. However, due to its low energy input and low exfoliation efficiency, LPE with bath sonication has little potential for scale-up production of GNPs. Recently, some researchers demonstrated that the production rate of GNPs can be raised substantially by high-power tip sonication [32–34] or combining tip sonication with shear mixing [35], and investigated the influences of vessel shape, initial graphite concentration, liquid volume, and surfactant on the yield of GNPs [33]. Moreover, Gao et al. presented a method to produce GNPs by exfoliating graphite in supercritical CO_2_/H_2_O medium via coupling a pressure reactor with a tip sonicator and investigated the effect of system pressure, sonication power, the ratio of supercritical CO_2_/H_2_O, etc. on the yield of graphene [36]. In addition, some researches proposed that the exfoliation efficiency and the quality of GNPs might be influenced by the sonication parameters, such as the input power, sonication time, probe diameter, and sonication frequency, etc. [14]. However, little research has been systematically conducted to understand the effect of tip sonication parameters on the quality of the produced GNPs.

This study aims to determine the effects of tip sonication power and time on the exfoliation of graphite into GNPs. First, a series of ethanol/water solvent mixtures with different surface tensions were used to disperse three kinds of flaked graphite samples. The solvent mixture with the highest GNP concentration was selected as the dispersing liquid medium. Then, the qualities of GNPs including their concentration, size, defect density, and sedimentation behavior, produced under different tip sonication powers and times were determined. The study has important implications for selecting the suitable tip sonication parameters in exfoliating graphite into GNPs.

## Methods/Experimental

### Selecting the Dispersing Liquid Medium

According to surface thermodynamics, the change of Gibbs free energy (Δ*G*) before and after graphite exfoliation can be used to predict the dispersion of GNPs. Generally, Δ*G* for exfoliating a piece of graphite into GNPs can be expressed as

1 $$ \Delta G=2N{\gamma}^{\mathrm{GL}}-2{\gamma}^{\mathrm{GL}}=2\left(N-1\right){\gamma}^{\mathrm{GL}} $$

where *N* is the number of GNPs after dispersion and *γ*^GL^ is the interfacial free energy between the GNPs and liquid medium. According to the combining rule, *γ*^GL^can be calculated from the surface tension of the GNPs (*γ*^GV^) and the surface tension of the liquid medium (*γ*^LV^), which can be expressed as

2 $$ {\gamma}^{\mathrm{GL}}={\gamma}^{\mathrm{GV}}+{\gamma}^{\mathrm{LV}}-2\sqrt{\gamma^{\mathrm{GV}}{\gamma}^{\mathrm{LV}}}={\left(\sqrt{\gamma^{\mathrm{GV}}}-\sqrt{\gamma^{\mathrm{LV}}}\right)}^2 $$

According to Eqs. (1) and (2), *γ*^GV^is constant, obviously, *γ*^LV^ affects the dispersion of GNPs, which has been indicated by some previous studies [11, 16]. In addition, it can be found that when *γ*^GV^ is equal to *γ*^LV^, Δ*G* is at its minimum, which indicates that it is favorable to disperse GNPs in the liquid medium.

Herein, to select a suitable dispersing liquid medium, a series of binary solvent mixtures with various surface tensions were prepared by mixing ethanol and ultrapure water with predefined ratios. The surface tensions of these solvent mixtures (ranging from 22 to 50 mJ/m^2^) were determined at 20 °C with a surface tensiometer (K100, Krüss GmbH, Germany). In the study, three commercially available flaked graphite samples with sizes of ~ 10 μm (denoted as G10; Xiamen Knano GNPs Technology Co. Ltd., China), ~ 30 μm (G30; Chengdu Organic Chemicals Co. Ltd., China), and ~ 100 μm (G100; Xiamen Knano GNPs Technology Co. Ltd., China) were used. During the experiment, the flaked graphite sample (4 mg) was added into a series of ethanol/water solvent mixtures (40 mL) and then exfoliated using a tip sonicator (Scientz-950E, Scientz Biotechnology Co. Ltd., China). The tip of sonicator had a diameter of 6 mm. The resulting GNP dispersion was centrifuged (TGL-10 K, Shanghai Anting Scientific Instrument, China) at 1000 rpm for 30 min to remove the aggregations. The concentrations of GNPs in a series of solvent mixtures with different surface tensions were measured by evaluating the optical density (OD) of each dispersion using an ultraviolet–visible (UV-Vis) spectrophotometer (Epoch, BioTek, Winooski, VT, USA). The solvent mixture with the highest GNP concentration was selected as the dispersing liquid medium for the following experiments.

### Graphite Exfoliation at Various Tip Sonication Parameters

To understand the effects of tip sonication power and time on the exfoliation behavior of graphite into GNPs, the flaked graphite samples were exfoliated by tip sonication at a power of 60, 100, 200, or 300 W for 10, 30, 60, 90, 120, or 180 min. In each exfoliation experiment, flaked graphite (4 mg) was added into the selected dispersing liquid medium (40 mL) and then sonicated by the tip sonicator. A temperature control system with a thermostatic water bath maintained the dispersion at 20 °C during sonication. The dispersion was centrifuged at 1000 rpm for 30 min to sediment the aggregated graphite flakes. Finally, the supernatant was collected to characterize the properties of GNPs produced under different tip sonication powers and times.

### Characterization of the Produced GNPs

To evaluate the quality of the GNPs produced using various tip sonication powers and times, the concentrations of GNP dispersions, the size, defect density, and layers of the GNPs, and sedimentation behavior of the GNPs in the selected dispersing liquid medium were characterized by various methods. Specifically, the size of the GNPs was observed by scanning electron microscopy (SEM; Nova NanoSEM 430, FEI, Hillsboro, OR, USA) at 10 kV. SEM samples were prepared by pipetting the GNP dispersions onto Si substrates. The defect density of the GNPs was characterized by Raman spectroscopy (LabRAM HR800, Horiba Jobin-Yvon, France) using a 514 nm laser. Samples for Raman spectroscopy were prepared by depositing GNP films onto glass slides. The concentrations of GNPs in the dispersions were measured by evaluating the OD of each dispersion at 600 nm using a UV-Vis spectrophotometer (Epoch, BioTek, Winooski, VT, USA). The sedimentation behavior of the GNPs in the selected dispersing liquid medium was estimated by determining the change of GNP concentration over time using the same UV-Vis spectrophotometer. The layers of the produced GNPs were determined by transmission electron microscopy (TEM; Tecnai F30, FEI, Hillsboro, OR, USA) at 200 kV. Samples for TEM analysis were prepared by pipetting each GNP dispersion onto a holey carbon mesh grid.

## Results and Discussion

### Exfoliating Graphite into GNPs in Liquid Media with Different Surface Tensions

**Fig. 1:**
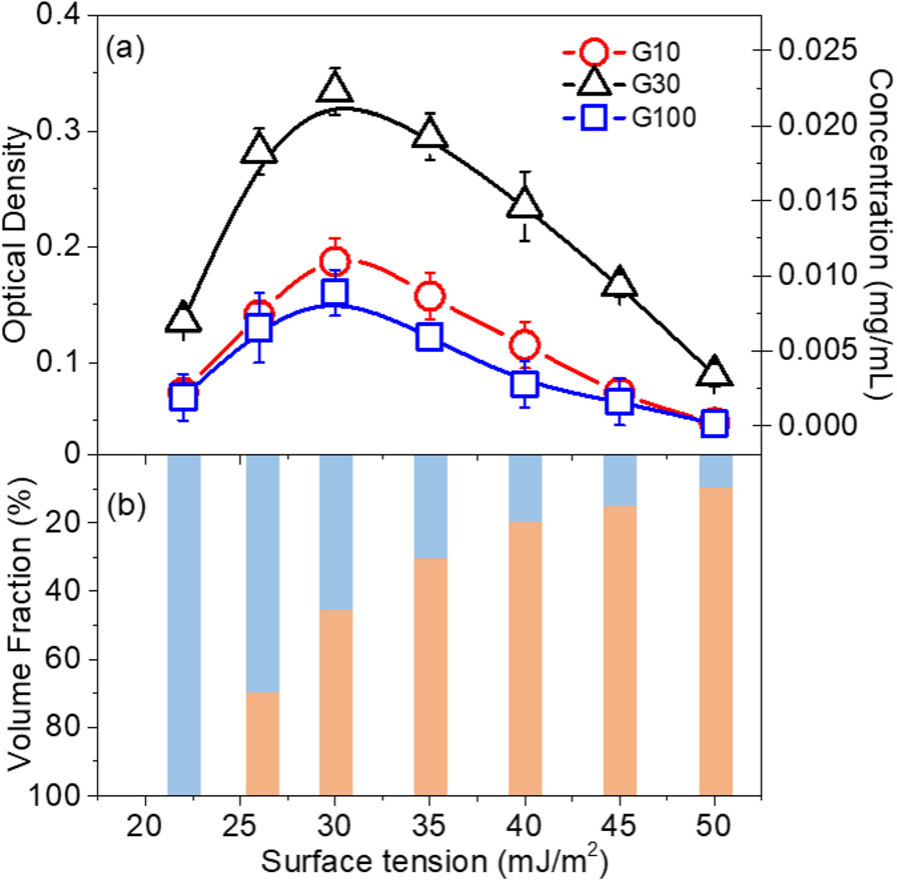
**a** Optical density and mass concentration of graphene dispersions produced by exfoliating G10, G30, and G100 flaked graphite samples as a function of the surface tension of ethanol-water solvent mixtures. **b** Relationships between the surface tension of solvent mixtures and the volume fractions of water (orange) and ethanol (blue)

Figure 1 shows the concentrations of GNPs in solvent mixtures of ethanol and ultrapure water with surface tensions ranging from 22 to 50 mJ/m^2^. In detail, the OD and mass concentration of the GNP dispersions as a function of the surface tension of the solvent mixtures are presented in Fig. 1a. In addition, the relationship between the mass concentration and OD of the GNP dispersions is shown in the Additional file 1. Figure 1b displays the relationship between the volume fraction of ethanol and surface tension of the solvent mixtures. The results indicated that the concentration of the GNP dispersions strongly depended on the surface tension of the solvent mixture. All three flaked graphite samples dispersed the most effectively in the ethanol (45 vol%)-water (55 vol%) mixture with a surface tension of ~ 30 mJ/m^2^, which was in good agreement with previous literature [17]. Therefore, the ethanol/water mixture with a surface tension of 30 mJ/m^2^ was selected as the dispersing liquid medium to exfoliate the flaked graphite samples.

### Concentrations of GNP Dispersion Produced Using Various Sonication Powers and Times

**Fig. 2:**
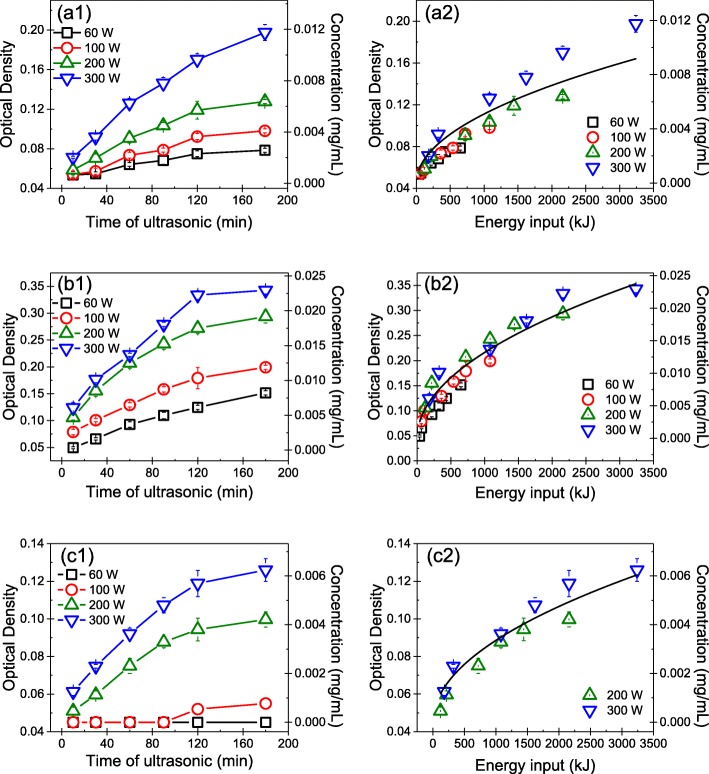
Concentrations of GNP dispersions produced by exfoliating (a1) G10, (b1) G30, and (c1) G100 using different sonication powers and times. The concentration of GNP dispersions produced by exfoliating (a2) G10, (b2) G30, and (c2) G100 as a function of sonication energy input

The concentrations of GNP dispersion produced using various sonication powers and times were determined by UV-Vis spectroscopy. Figure 2(a1), (b1), and (c1) shows the OD and mass concentration of the GNPs produced in the water-ethanol mixture with a surface tension of 30 mJ/m^2^ as functions of the sonication power and time. The results indicated that the concentration of GNP dispersions increased with both the sonication power and time. Note that, G100 was not exfoliated in the ethanol-water mixture with a surface tension of 30 mJ/m^2^ at sonication powers of 60 and 100 W. Specifically, for the same sonication time, the concentration of GNP dispersions increased with sonication power. Furthermore, at the same sonication power, the concentration of the GNP dispersions increased rapidly at first and then more slowly as the sonication time lengthened. Once the sonication time reached 120 min, the concentration of GNP dispersions remained almost unchanged. These results indicated that the maximum concentration of GNP dispersions was obtained after a certain sonication time, after which further sonication was not effective. Moreover, the results demonstrated that the concentrations of GNP dispersions produced at a sonication power of 300 W were much higher than those of dispersions produced at sonication powers of 60, 100, and 200 W.

To evaluate the influence of tip sonication power and time on the concentration of the GNP dispersions, the relationship between the energy input, i.e., *E*, (sonication time multiplied by sonication power) and concentration of the GNP dispersions was determined. Figure 2(a2), (b2), and (c2) reveals that the relationship between the concentration of the GNP dispersions and energy input can be described by $$ {C}_g={aE}^{\raisebox{1ex}{$1$}\!\left/ \!\raisebox{-1ex}{$2$}\right.} $$, where *C*_*g*_ is the concentration of the GNP dispersion, and *a* is a parameter determined by fitting the experimental data. The *a* values for the GNP dispersions obtained by exfoliating G10, G30, and G100 are 1.612 × 10^− 4^, 4.175 × 10^− 4^, and 1.061 × 10^− 4^ mg/(mL · kJ^½^), respectively. These results demonstrated that with increasing energy input, the concentration of the GNP dispersion increased rapidly at first and then slowly, which was in good agreement with the previous findings of Coleman [23] and Bracamonte [37] on exfoliating graphite into GNPs via bath sonication.

### Size of GNPs Produced Using Various Sonication Powers and Times

**Fig. 3:**
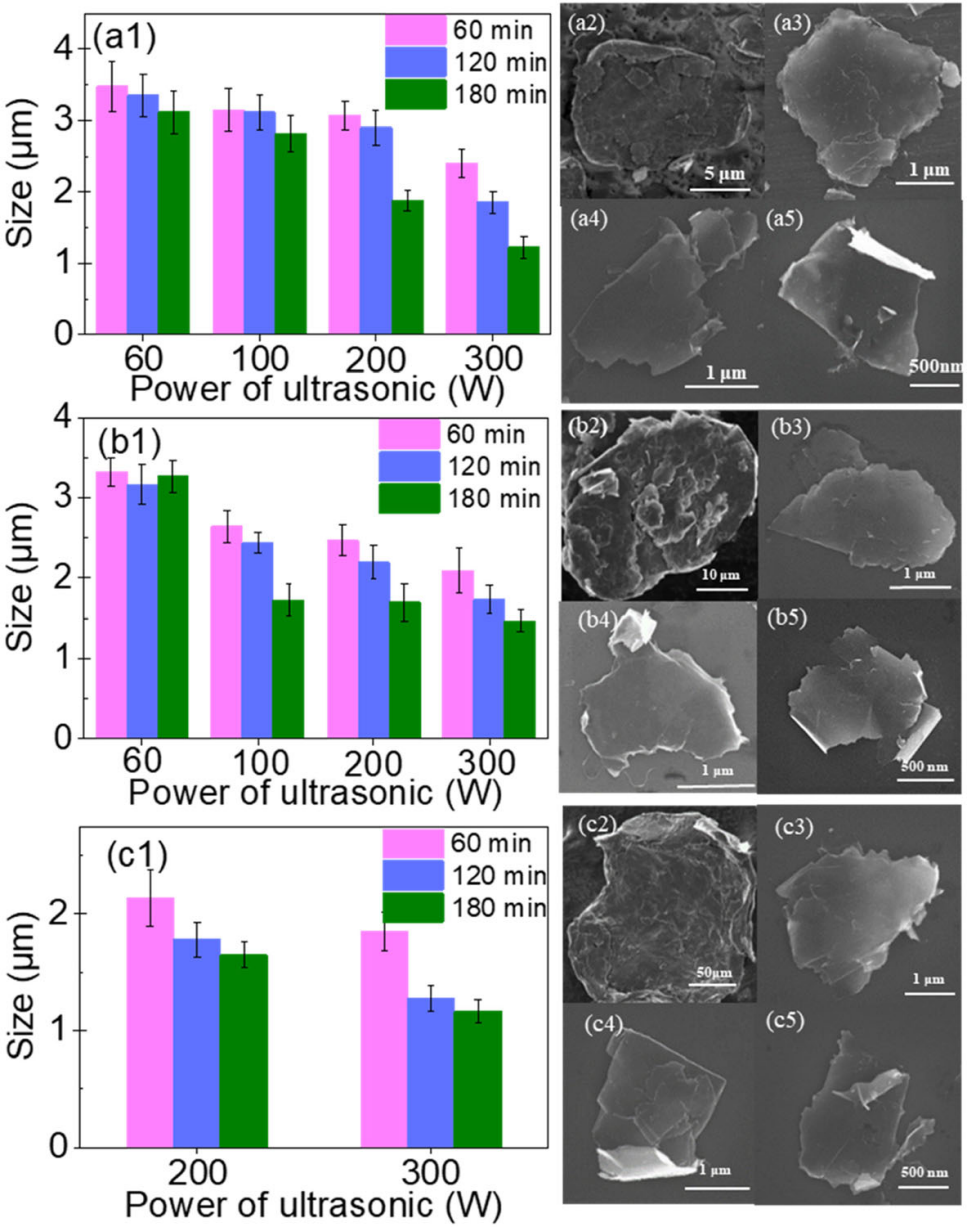
Mean size of GNPs produced using different sonication powers and times to exfoliate (a1) G10, (b1) G30, and (c1) G100 flaked graphite samples. SEM images of the flaked graphite samples (a2) G10, (b2) G30, and (c2) G100. SEM images of the GNPs produced by exfoliating G10 at a sonication power of 300 W for (a3) 60 min, (a4) 120 min, and (a5) 180 min. SEM images of the GNPs produced by exfoliating G30 at a sonication power of 300 W for (b3) 60 min, (b4) 120 min, and (b5) 180 min. SEM images of the GNPs produced by exfoliating G100 at a sonication power of 300 W for (c3) 60 min, (c4) 120 min, and (c5) 180 min

Figure 3 shows the size of GNPs produced by exfoliating G10, G30, and G100 flaked graphite samples using various tip sonication powers and times. Figure 3(a1), (b1), and (c1) displays the mean size of GNPs produced by exfoliating G10, G30, and G100 using different tip sonication powers and times. The mean size of GNPs was determined by analyzing about 100 GNPs for each sample. The results indicated that with increasing sonication power and time, the size of the produced GNPs decreased slightly. Regardless of the initial size of the flaked graphite, the size of the GNPs produced using various tip sonication times and powers ranged from ~ 1 to ~ 3 μm. Because G100 was not exfoliated at sonication powers of 60 and 100 W, Fig. 3(c1) only shows the size of GNPs exfoliated at sonication powers of 200 and 300 W.

To illustrate the influence of sonication on the size of produced GNPs, SEM images of these three graphite samples and GNPs obtained by exfoliation at a sonication power of 300 W for different periods are provided in Fig. 3. The SEM images of GNPs exfoliated at a tip sonication power of 60, 100, and 200 W for 60, 120, and 180 min are shown in the Additional file 1. Specifically, Fig. 3(a2), (b2), and (c2) shows the initial sizes of the graphite flakes, i.e., G10, G30, and G100, respectively. The images indicate that G10, G30, and G100 were all many layers thick and have sizes of approximately 10, 30, and 100 μm, respectively. Figure 3(a3), (a4), and (a5) depicts SEM images of GNPs produced by exfoliating G10 in ethanol-water mixtures at a tip sonication power of 300 W for 60, 120, and 180 min, respectively. It can be found that when the sonication time was 60 min, the produced GNPs were a little thicker than those obtained by sonication for 120 or 180 min; the latter two sonication times gave GNPs of almost the same thickness. Figure 3(b3), (b4), and (b5) display SEM images of GNPs produced by exfoliating G30 at a tip sonication power of 300 W for 60, 120, and 180 min, respectively. Meanwhile, Fig. 3(c3), (c4), and (c5) shows SEM images of GNPs produced by exfoliating G100 at a tip sonication power of 300 W for 60, 120, and 180 min, respectively. These results all indicated that with increasing sonication power and time, the thickness of the produced GNPs decreased.

Overall, the results demonstrated that with increasing sonication power and time, the size of the produced GNPs decreased slightly. However, regardless of the initial size of the flaked graphite, the GNPs produced using various tip sonication times and powers ranged from ~ 1 to ~ 3 μm in size.

### Defect Density of GNPs Produced Using Various Sonication Powers and Times

**Fig. 4:**
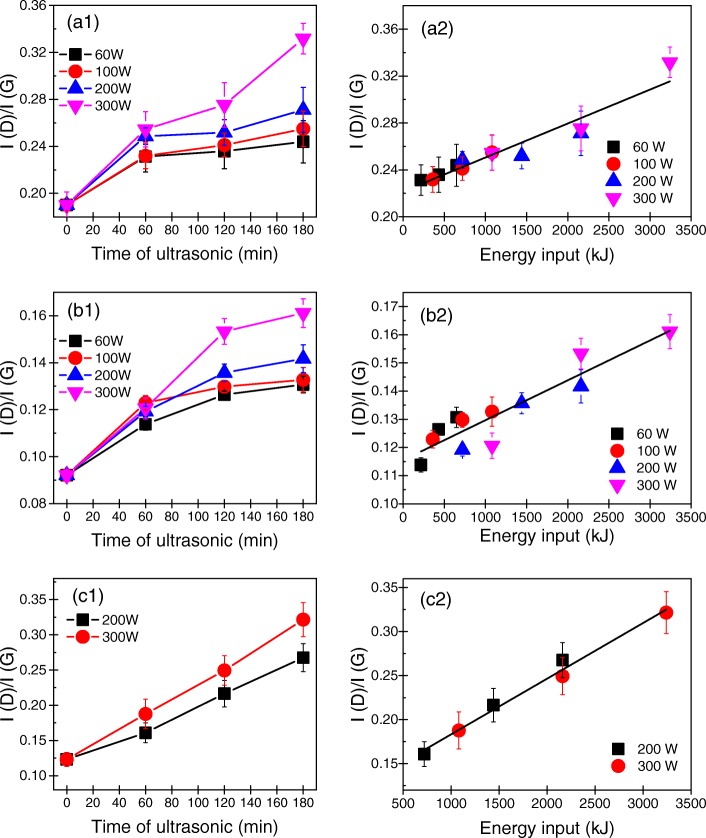
*I*_D_/*I*_G_ values of the GNPs produced using various sonication powers and times from (a1) G10, (b1) G30, and (c1) G100. *I*_D_/*I*_G_ values of the GNPs produced by exfoliating (a2) G10, (b2) G30, and (c2) G100 as a function of sonication energy input

The defect density of GNPs produced using various sonication powers and times were determined by Raman spectroscopy. Generally, the ratio of intensity of the *D* band at 1350 cm^− 1^ to that of the *G* band at 1580 cm^− 1^ (*I*_D_/*I*_G_) is used to characterize the defect density of GNPs [33]. A smaller *I*_D_/*I*_G_ value indicates a lower defect density of the GNPs. The *I*_D_/*I*_G_ values of the GNPs exfoliated using different sonication powers and times are shown as histograms in Fig. 4(a1), (b1), and (c1). In addition, typical Raman spectra of the initial graphite flakes and GNPs exfoliated at powers of 60, 100, 200, or 300 W for 60, 120, or 180 min are presented in the Additional file 1. The *I*_D_/*I*_G_ values of the GNPs rose slightly with increasing tip sonication time and power. Nevertheless, the *I*_D_/*I*_G_ values of GNPs produced using the various tip sonication powers and times ranged from ~ 0.1 to ~ 0.3, which indicated that all the produced GNPs had a low defect density, that is, they were of high quality. In addition, Additional file 1: Figures S5, S6, and S7 illustrate that with increasing sonication power and time, the *G* bands of the GNPs became broader, which meant that most defects in the GNPs were edge defects rather than basal plane defects.

To thoroughly understand the influence of tip sonication power and time on the defect density of the produced GNPs, the energy input during exfoliation was considered. Figure 4(a2), (b2), and (c2) shows the relationships between the *I*_D_/*I*_G_ value and energy input during tip sonication. Evidently, regardless of the initial size of the flaked graphite sample, *I*_D_/*I*_G_ of all the produced GNPs increased linearly with the energy input. It indicated that to produce high-quality GNPs, the sonication power and time should be decreased. In addition, the results showed that the *I*_D_/*I*_G_ values of the GNPs produced by exfoliating G30 were much lower than those of GNPs produced by exfoliating G10 and G100. This may be caused by differences in the quality of the pristine graphite samples.

### Sedimentation Behavior of GNPs in a Liquid Medium

**Fig. 5:**
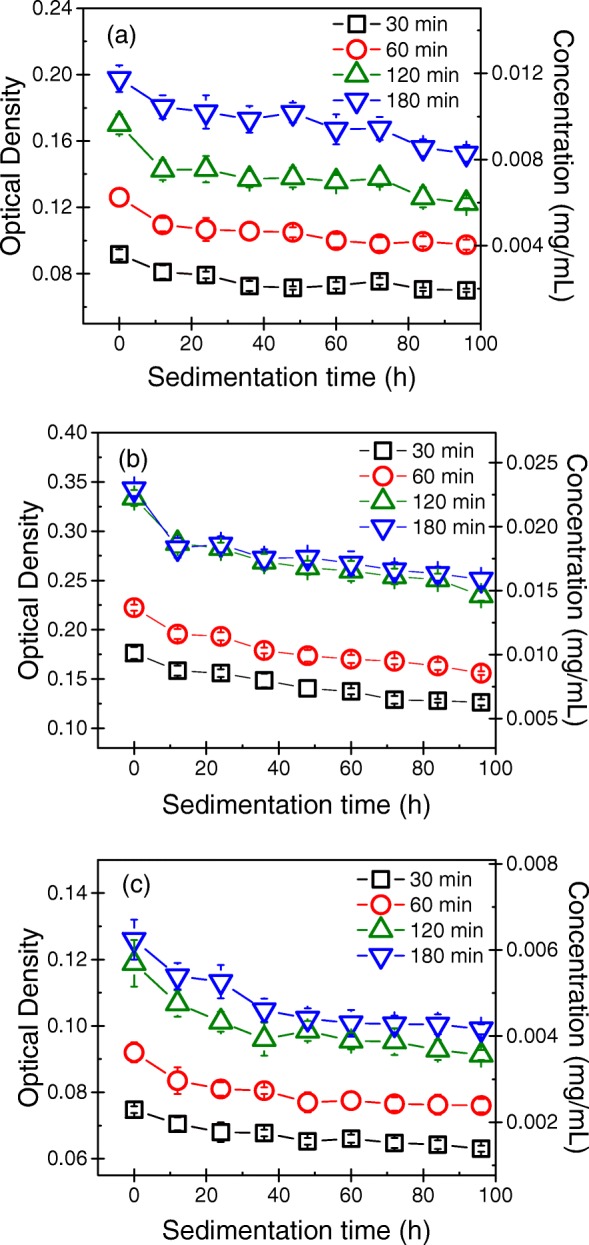
Sedimentation curves of the GNP dispersions produced by exfoliating **a** G10, **b** G30, and **c** G100 at a sonication power of 300 W

The sedimentation behavior of the GNPs in a liquid medium represents the stability of the graphene dispersion. Figure 5 illustrates the sedimentation behavior of the GNPs in liquid media produced at a sonication power of 300 W for 30, 60, 120, and 180 min estimated by determining the OD of the GNP dispersions as a function of sedimentation time. The sedimentation curves for GNP dispersions produced at sonication powers of 60, 100, and 200 W for 30, 60, 120, and 180 min can be found in Additional file 1. The results indicated that the concentrations of the GNP dispersions produced using different sonication powers and times all decreased rapidly over the first 12 h and then leveled off. After sedimentation for 96 h, the concentrations of the GNP dispersions produced by exfoliating G10 at a sonication power of 300 W for 60, 120, and 180 min were 61.8%, 70.1%, and 70.5% of their initial concentrations, respectively. For G30, after sedimentation for 96 h, the concentrations of the GNP dispersions produced using a sonication power of 300 W for 60, 120, and 180 min were 62.5%, 71.2%, and 71.2% of the initial concentration of the corresponding GNP dispersions, respectively. Meanwhile, after sedimentation for 96 h of the GNP dispersions produced from G100 using a sonication power of 300 W for 60, 120, and 180 min, the concentrations of the dispersions were 65.9%, 71.6%, and 72.3% of their initial values, respectively.

These results indicated that the concentrations of the GNP dispersions produced using different sonication powers and times all decreased rapidly over the first 12 h and then leveled off. After sedimentation for 96 h, the concentrations of the GNP dispersions were approximately 70% of their initial values. In addition, the stabilities of the GNP dispersions in liquid media produced at various sonication powers for 120 min were almost the same as those produced at various sonication powers for 180 min.

### Implications for Selecting the Suitable Tip Sonication Parameters

Based on the quality of GNPs exfoliated using various tip sonication powers and times, it can be found that the size of the GNPs ranged from ~ 1 to ~ 3 μm regardless of the initial size of the flaked graphite. Meanwhile, the *I*_D_/*I*_G_ values of the GNPs produced using various tip sonication powers and times showed that all the GNPs were of high quality. Furthermore, the concentrations of GNP dispersions produced at a sonication power of 300 W were much higher than those of the dispersions produced at sonication powers of 60, 100, and 200 W. In addition, the sedimentation curves of the GNP dispersions indicated that the stabilities of the GNP dispersions produced at various sonication powers for 120 min were almost the same as those of the dispersion produced at various sonication powers for 180 min. Taking into account all of the above-mentioned factors, we think that the suitable tip sonication parameters to exfoliate graphite to form GNPs might be the sonication power of 300 W for 120 min.

**Fig. 6:**
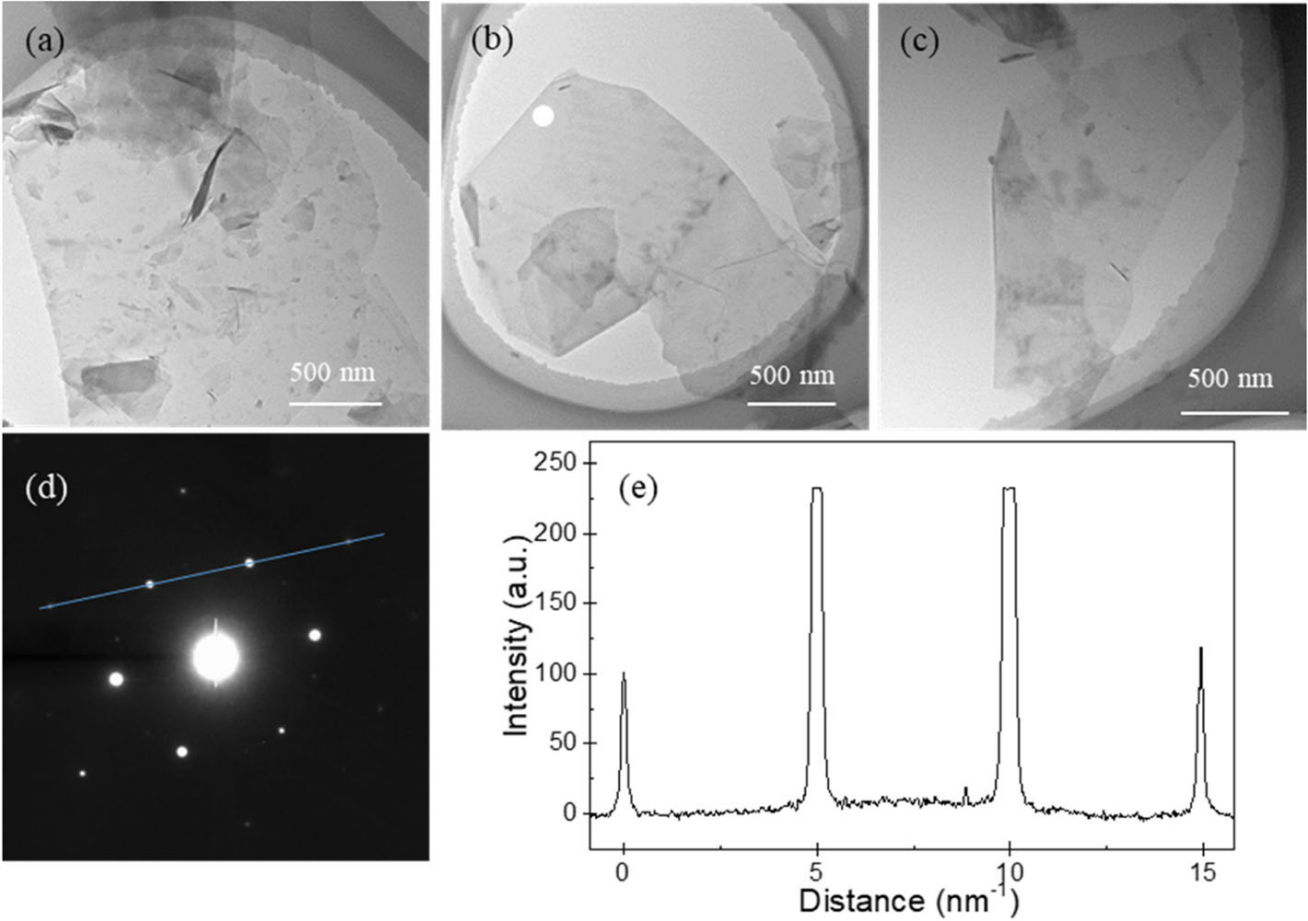
TEM images of GNPs produced by exfoliating **a** G10, **b** G30, and **c** G100 flaked graphite samples at a sonication power of 300 W for 120 min. **d** Electron diffraction pattern taken from the position of the white circle in **b**. **e** Diffraction intensity taken along the line in **d**

Moreover, the thickness of GNPs is generally an important indicator of their quality. Therefore, the thickness of the GNPs produced by sonication at 300 W for 120 min was further determined by TEM. Figure 6a–c shows the bright-field TEM images of GNPs produced by exfoliating G10, G30, and G100 at a sonication power of 300 W for 120 min, respectively. To identify the presence of monolayer or few-layer GNPs produced at a sonication power of 300 W for 120 min, an electron diffraction pattern of the GNPs was measured at an incidence angle of 0°. Specifically, Fig. 6d shows an electron diffraction pattern of the GNP in Fig. 6b, which contains a six-fold symmetry pattern consistent with the typical crystal structure of a GNP. In addition, in this hexagonal pattern, the intensity of the {1100} points is stronger than that of the {2110} points. To inspect the ratio of the intensity {1100} to that of {2110} (*I*_{1100}_/*I*_{2110}_), some of the points were fitted by a line, as shown in Fig. 6d. Figure 6e reveals that the inner peaks are more intense than the outer ones and *I*_{1100}_/*I*_{2110}_ is approximately 1.30. Previous work showed that when *I*_{1100}_/*I*_{2110}_ < 1, the GNP should be multilayer with AB stacking, whereas when *I*_{1100}_/*I*_{2110}_ > 1, the GNP should be monolayer [38]. Therefore, the results indicated that monolayer or few-layer GNPs were produced using a sonication power of 300 W for 120 min.

## Conclusions

The influence of tip sonication power and time on the exfoliation of graphite into GNPs was determined by analyzing the concentration of GNP dispersions, the size and defect density of the produced GNPs, and sedimentation behavior of GNP dispersions. The results indicated that the concentration of the GNP dispersions was related to the product of sonication power and time, i.e., sonication energy input. The relationship between the concentration of a GNP dispersion and sonication energy input can be described by $$ {C}_g={aE}^{\raisebox{1ex}{$1$}\!\left/ \!\raisebox{-1ex}{$2$}\right.} $$. With the increase of sonication power and time, the size of the produced GNPs decreased, while, the defect density of GNPs increased slightly. The sedimentation curves of the GNP dispersions indicated that the concentrations of all the GNP dispersions were approximately 70% of their initial values, after sedimentation for 96 h. The TEM images indicated that the GNPs exfoliated under sonication power of 300 W for 120 min were few-layer. The study has important implications for selecting the suitable tip sonicating parameters in exfoliating graphite into GNPs.

## Additional file

Additional file 1: **Figure S1.** Relationship between the optical density (OD) and concentration of graphene nanoplatelets (GNPs) dispersions. **Figure S2.** SEM images of GNPs produced by exfoliating G10 at sonication powers of 60 W (row a), 100 W (row b), and 200 W (row c) for 60 min (column 1), 120 min (column 2), and 180 min (column 3). **Figure S3.** SEM images of GNPs produced by exfoliating G30 at sonication powers of 60 W (row a), 100 W (row b), and 200 W (row c) for 60 min (column 1), 120 min (column 2), and 180 min (column 3). **Figure S4.** SEM images of GNPs produced by exfoliating G100 at a sonication power of 200 W for (a) 60 min, (b) 120 min, and (c) 180 min. **Figure S5.** Raman spectra of pristine G10 and GNPs produced by exfoliating G10 at various powers for (a) 60 min, (b) 120 min, and (c) 180 min. **Figure S6.** Raman spectra of pristine G30 and GNPs produced by exfoliating G30 at various powers for (a) 60 min, (b) 120 min, and (c) 180 min. **Figure S7.** Raman spectra of pristine G100 and GNPs produced by exfoliating G100 at various powers for (a) 60 min, (b) 120 min, and (c) 180 min. **Figure S8.** Sedimentation curves of GNP dispersions produced by exfoliating G10 at sonication powers of (a) 60 W, (b) 100 W, and (c) 200 W. **Figure S9.** Sedimentation curves of GNP dispersions produced by exfoliating G30 at sonication powers of (a) 60 W, (b) 100 W, and (c) 200 W. Figure S10. Sedimentation curves of GNP dispersions produced by exfoliating G100 at a sonication power of 200 W for different periods. (DOCX 2559 kb)

## Abbreviations

CVD: Chemical vapor deposition
GNPS: Graphene nanoplatelets
LPE: Liquid-phase exfoliation
OD: Optical density
SEM: Scanning electron microscopy
TEM: Transmission electron microscopy
