# Supplementary material for: Effects of Tip Sonication Parameters on Liquid Phase Exfoliation of Graphite into Graphene Nanoplatelets
Source: Nanoscale Res Lett. 2018 Aug 17;13:241. doi: 10.1186/s11671-018-2648-5 (PMC6097981; doi:10.1186/s11671-018-2648-5)
Supplement: Supplementary file 1 — Figure S1. Relationship between the optical density (OD) and concentration of graphene nanoplatelets (GNPs) dispersions. Figure S2. SEM images of GNPs produced by exfoliating G10 at sonication powers of 60 W (row a), 100 W (row b), and 200 W (row c) for 60 min (column 1), 120 min (column 2), and 180 min (column 3). Figure S3. SEM images of GNPs produced by exfoliating G30 at sonication powers of 60 W (row a), 100 W (row b), and 200 W (row c) for 60 min (column 1), 120 min (column 2), and 180 min (column 3). Figure S4. SEM images of GNPs produced by exfoliating G100 at a sonication power of 200 W for (a) 60 min, (b) 120 min, and (c) 180 min. Figure S5. Raman spectra of pristine G10 and GNPs produced by exfoliating G10 at various powers for (a) 60 min, (b) 120 min, and (c) 180 min. Figure S6. Raman spectra of pristine G30 and GNPs produced by exfoliating G30 at various powers for (a) 60 min, (b) 120 min, and (c) 180 min. Figure S7. Raman spectra of pristine G100 and GNPs produced by exfoliating G100 at various powers for (a) 60 min, (b) 120 min, and (c) 180 min. Figure S8. Sedimentation curves of GNP dispersions produced by exfoliating G10 at sonication powers of (a) 60 W, (b) 100 W, and (c) 200 W. Figure S9. Sedimentation curves of GNP dispersions produced by exfoliating G30 at sonication powers of (a) 60 W, (b) 100 W, and (c) 200 W. Figure S10. Sedimentation curves of GNP dispersions produced by exfoliating G100 at a sonication power of 200 W for different periods. (DOCX 2559 kb) [file 11671_2018_2648_MOESM1_ESM.docx]

**Additional file 1**

**Effects of tip sonication parameters on liquid phase exfoliation of graphite into graphene nanoplatlets**

*Xinzhi Cai, ^a^ Zeyi Jiang, ^a,^* *^b^ Xinru Zhang,* *^a,^* *^c^* ^*^*Xinxin Zhang ^a, c^*

^a^ School of Energy and Environmental Engineering, University of Science and Technology Beijing, Beijing, 100083, China

^b^ Beijing Key Laboratory for Energy Saving and Emission Reduction of Metallurgical Industry, University of Science and Technology Beijing, Beijing, 100083, China

^c^ Beijing Engineering Research Center of Energy Saving and Environmental Protection, University of Science and Technology Beijing, Beijing, 100083, China

**Corresponding Author**

*Xinru Zhang

Mailing Address: School of Energy and Environmental Engineering, University of Science and Technology Beijing, Beijing, 100083, China

Phone: 86-10-62334971; E-mail: [xinruzhang@ustb.edu.cn](mailto:xinruzhang@ustb.edu.cn)


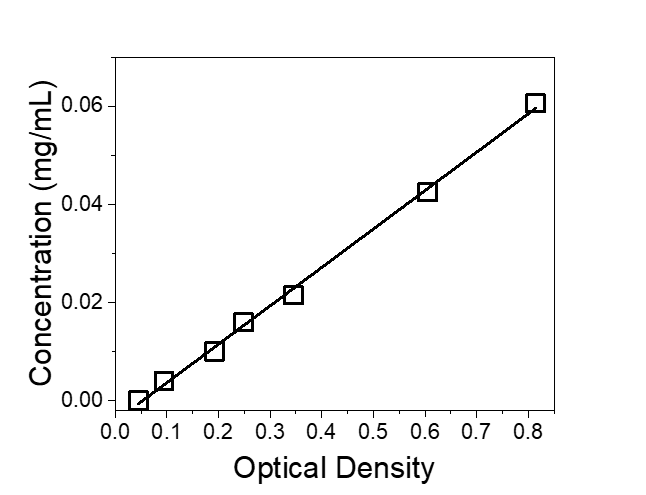


**Figure S1.** Relationship between the optical density (OD) and concentration of graphene nanoplatelets (GNPs) dispersions.


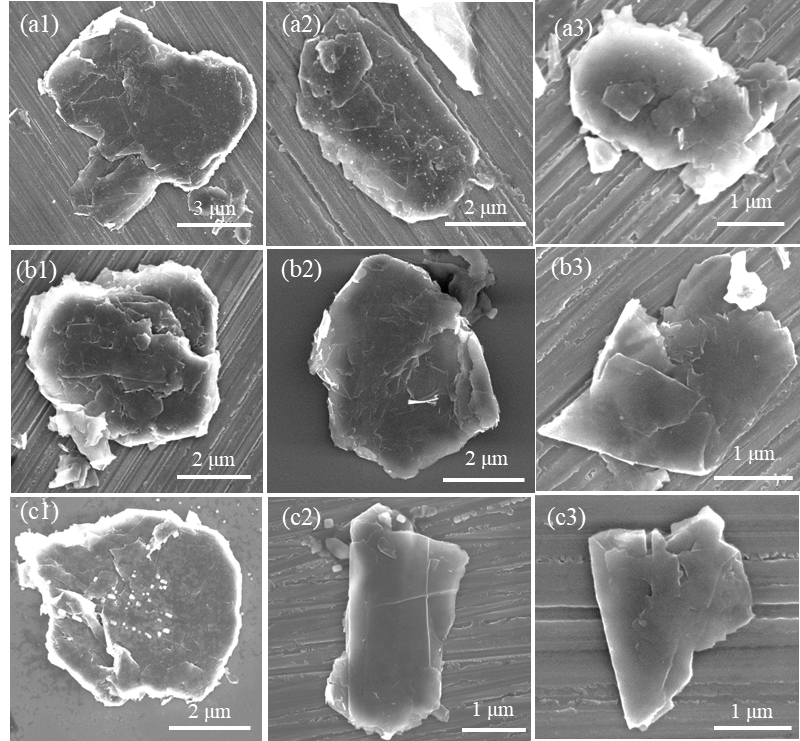


**Figure S2.** SEM images of GNPs produced by exfoliating G10 at sonication powers of 60 W (row a), 100 W (row b), and 200 W (row c) for 60 min (column 1), 120 min (column 2), and 180 min (column 3).


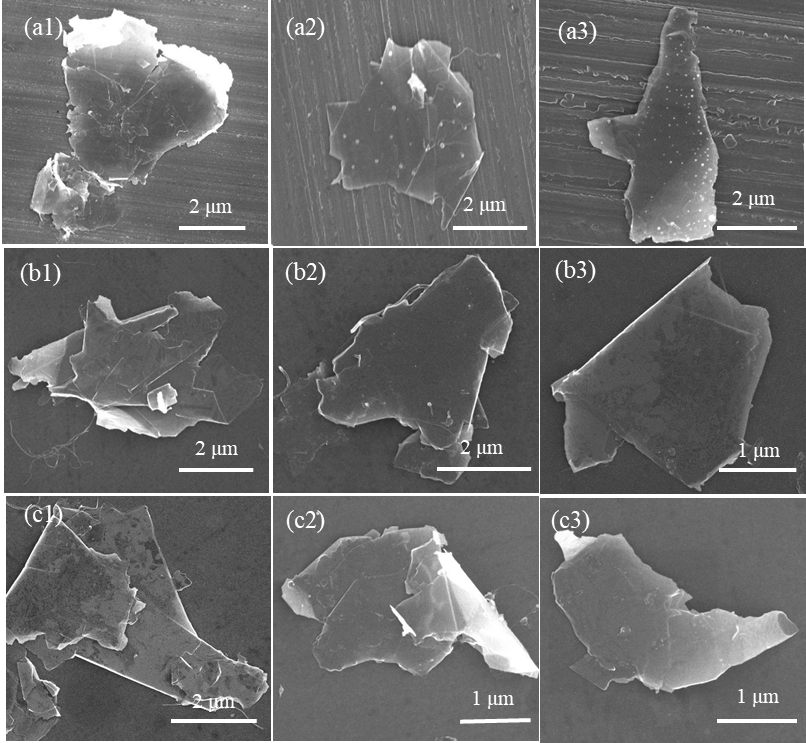


**Figure S3.** SEM images of GNPs produced by exfoliating G30 at sonication powers of 60 W (row a), 100 W (row b), and 200 W (row c) for 60 min (column 1), 120 min (column 2), and 180 min (column 3).


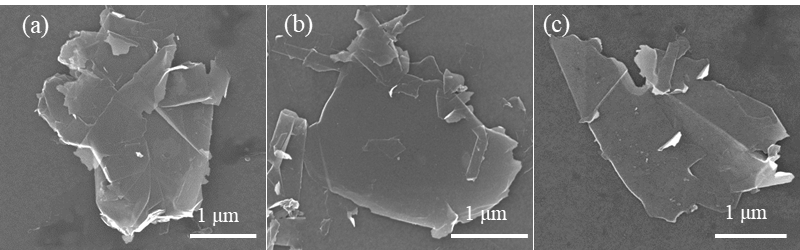


**Figure S4.** SEM images of GNPs produced by exfoliating G100 at a sonication power of 200 W for (a) 60 min, (b) 120 min, and (c) 180 min.

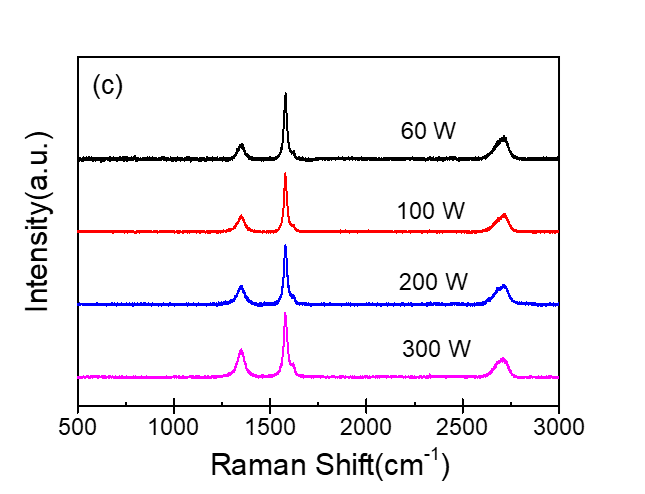


**Figure S5.** Raman spectra of pristine G10 and GNPs produced by exfoliating G10 at various powers for (a) 60 min, (b) 120 min, and (c) 180 min.


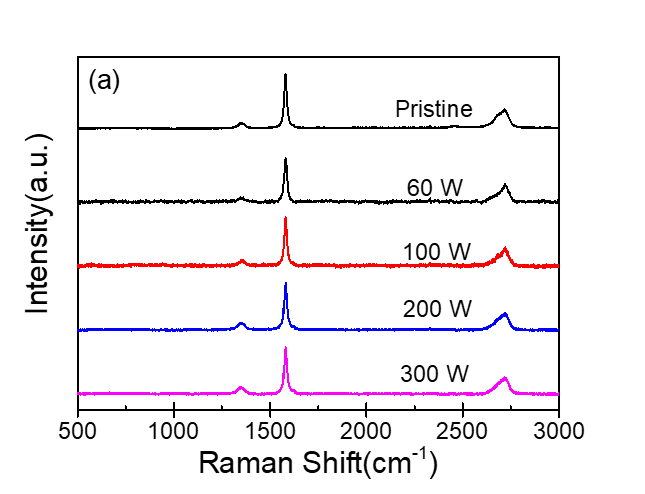


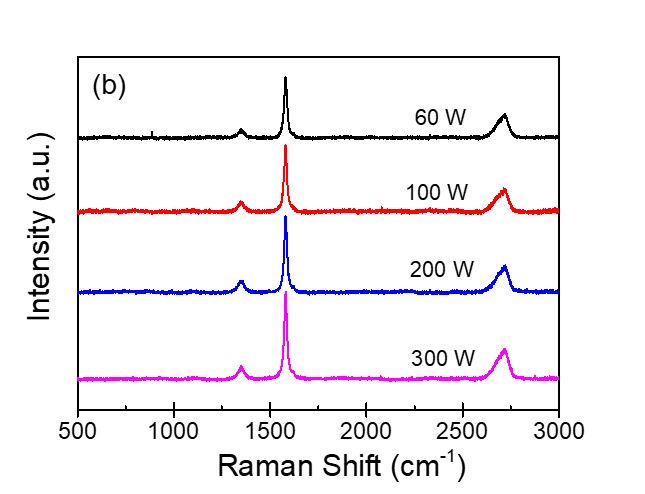


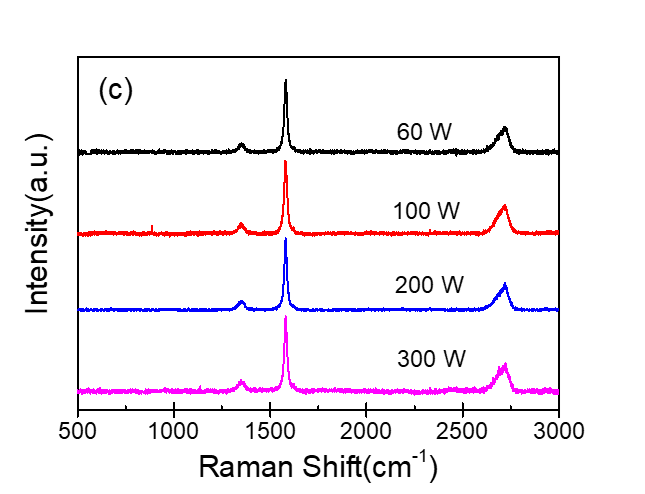


**Figure S6.** Raman spectra of pristine G30 and GNPs produced by exfoliating G30 at various powers for (a) 60 min, (b) 120 min, and (c) 180 min.

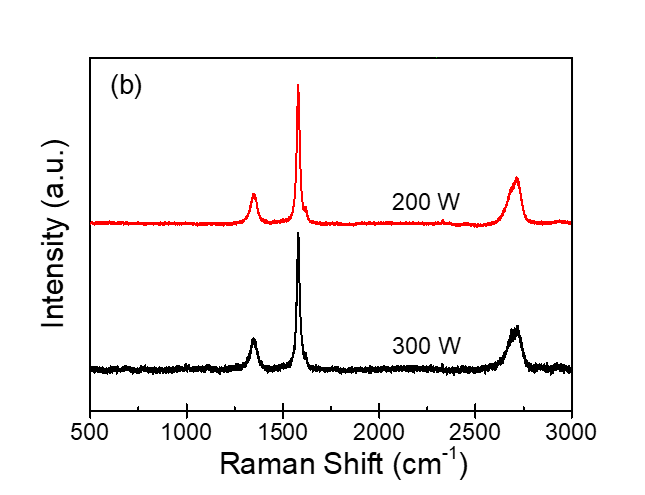


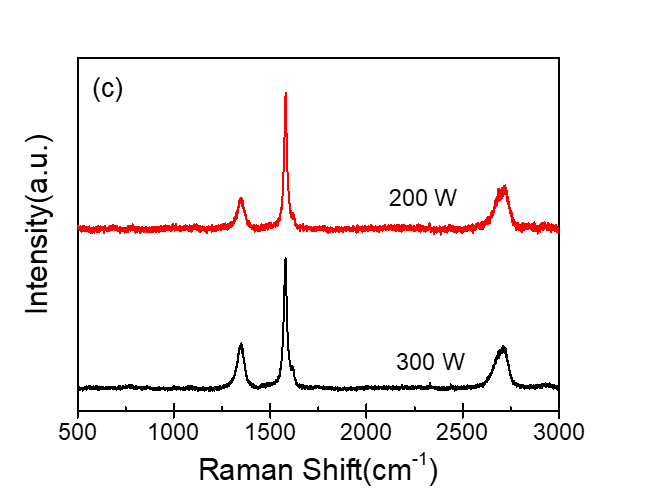


**Figure S7.** Raman spectra of pristine G100 and GNPs produced by exfoliating G100 at various powers for (a) 60 min, (b) 120 min, and (c) 180 min.

**Figure S8.** Sedimentation curves of GNP dispersions produced by exfoliating G10 at sonication powers of (a) 60 W, (b) 100 W, and (c) 200 W.

**Figure S9.** Sedimentation curves of GNP dispersions produced by exfoliating G30 at sonication powers of (a) 60 W, (b) 100 W, and (c) 200 W.

**Figure S10.** Sedimentation curves of GNP dispersions produced by exfoliating G100 at a sonication power of 200 W for different periods.
